# Investigating bottom-up auditory attention

**DOI:** 10.3389/fnhum.2014.00327

**Published:** 2014-05-27

**Authors:** Emine Merve Kaya, Mounya Elhilali

**Affiliations:** Department of Electrical and Computer Engineering, The Johns Hopkins UniversityBaltimore, MD, USA

**Keywords:** audition, attention, saliency, bottom-up, psychoacoustics

## Abstract

Bottom-up attention is a sensory-driven selection mechanism that directs perception toward a subset of the stimulus that is considered salient, or attention-grabbing. Most studies of bottom-up auditory attention have adapted frameworks similar to visual attention models whereby local or global “contrast” is a central concept in defining salient elements in a scene. In the current study, we take a more fundamental approach to modeling auditory attention; providing the first examination of the space of auditory saliency spanning pitch, intensity and timbre; and shedding light on complex interactions among these features. Informed by psychoacoustic results, we develop a computational model of auditory saliency implementing a novel attentional framework, guided by processes hypothesized to take place in the auditory pathway. In particular, the model tests the hypothesis that perception tracks the evolution of sound events in a multidimensional feature space, and flags any deviation from background statistics as salient. Predictions from the model corroborate the relationship between bottom-up auditory attention and statistical inference, and argues for a potential role of predictive coding as mechanism for saliency detection in acoustic scenes.

## 1. Introduction

Sounds in everyday life seldom appear in isolation. We are constantly flooded with a cacophony of sounds that impinge on our ears at every instant. Our auditory system is tasked with sorting through this sensory flow, to attend to and identify sound objects of interest; all while ignoring irrelevant distracters and ambient backgrounds—a phenomenon referred to as the “cocktail party effect” (Cherry, 1953). A key process in parsing acoustic scenes is the role of attention, which mediates perception and behavior by focusing both sensory and cognitive resources on pertinent information in the stimulus space. At a cocktail party, we can tune out surrounding sounds to listen to one specific conversation, but the shattering sound of a waiter dropping a tray of glasses will nonetheless cause us to pause to attend to the unexpected event.

Attention is not a monolithic process (Driver, 2001). It can be modulated by “bottom-up” sensory-driven factors, “top-down” task-specific goals, expectations, and learned schemas; as well as “lateral-based” behavioral history and reward (Awh et al., 2012). It refers to a process or group of processes that act as selection mechanisms and allow the sensory and perceptual systems to form a processing bottleneck or focus cognitive resources on a subset of incoming stimuli deemed interesting. In the case of purely “bottom-up” attention, the selection process is driven by sensory cues that orient our attention to interesting events in the environment. It is guided by inherent properties of an event that cause it to stand out with respect to surrounding sounds, regardless of the listener's goal or task at hand.

Some stimuli are inherently conspicuous and pop out amidst certain backgrounds. The study of bottom-up attentional effects is ultimately an investigation of physical attributes of sensory space and integrative mechanisms that allow regions of this space to become salient. In vision, bottom-up attention has been likened to a contrast match concept (Itti and Koch, 2001). Visual elements that differ along modalities of color, intensity, orientation, size and depth (among others) are shown to affect visual search (Wolfe and Horowitz, 2004), and bias eye fixations in natural scenes (Masciocchi et al., 2009). The synergy between the physical structure of a visual scene and saliency-based selective visual attention is a complex one (Wolfe et al., 2011); but has nonetheless been translated into successful mathematical implementations (Borji et al., 2013a) based on contrast analysis of spatial scales (Itti et al., 1998), local geometry (Seo and Milanfar, 2009), or spectral contrast (Hou and Zhang, 2007; Li et al., 2012) using a variety of measures including information entropy (Bruce and Tsotsos, 2009) and natural statistics (Zhang et al., 2008). Similar approaches have been explored in the auditory modality with limited success. Adaptations of the visual saliency map have been introduced by considering the time-frequency spectrogram of an audio signal as an “auditory image” upon which saliency mechanisms can operate (Kayser et al., 2005). This architecture has also been extended to extract attributes better suited for the auditory domain such as a pitch (Duangudom and Anderson, 2007; Kalinli and Narayanan, 2007). However, these models remain constrained by the limitations imposed by the visual domain in computing within-feature and across-feature competition for attention; limitations that do not exist in the auditory domain (Ihlefeld and Shinn-Cunningham, 2008). The nature of sound as a time-evolving entity cannot be captured by spatial processing. There have been attempts to remedy this problem by changes to the procedure of computing saliency after feature extraction, but the methodologies used are still adaptations from vision mechanisms (Kaya and Elhilali, 2012; Cottrell and Tsuchida, 2012). In this work, we discard the traditional framework of computing a spatial saliency map, and employ psychoacoustical experimentation and computational modeling to build a saliency extraction mechanism that broadly mimics processes that are hyphothesized to take place in the auditory pathway.

Although no evidence has been found for a dedicated auditory saliency map in the brain, the well researched mechanisms of deviance detection in the auditory pathway could be potentially implicated in the perception of saliency in audition. The neural correlates of these mechanisms have long been investigated, leading to the birth of multiple theories (Naatanen et al., 1978; May and Tiitinen, 2010). The recent theory of “predictive coding” (Winkler, 2007) provides a unifying framework to encompass some of the previously competing theories under the umbrella of an overall Bayesian brain hypothesis (Knill and Pouget, 2004; Friston, 2005). The Bayesian brain uses generative models to predict sensory input, adjusting its internal probabilistic representations based on novel sensory information. In this setup, predictive coding corresponds to minimizing error between bottom-up sensations and top-down predictions, with the corresponding mismatch signaling the detection of a deviant. There has been considerable support for the theory of prediction-based deviance detection in the auditory domain as the best explanation of neurophysiological observations from electroencephalography (EEG) studies employing simple repeating tones and sound patterns (Winkler, 2007; Garrido et al., 2009). However, there has been no proposal of an explicit tie between this framework and bottom-up attention in complex natural soundscapes. In this work, we aim to bridge this gap by asking whether the predictive-coding theory can provide an explanation for auditory saliency. To this end, we define a salient auditory event as one that deviates from the feature regularities in the sounds preceding it. In the cocktail party example, the salient shattering glasses would differ from the ambient sounds in acoustic attributes such as timbre, intensity, and location.

We conduct human behavioral experiments to gain psychophysiological insight into the dimensions of auditory saliency and their interactions. In the visual domain, the primary method of obtaining a human ground-truth for the saliency measure is to record eye movements while free-viewing images (Parkhurst et al., 2002; Tatler et al., 2011). However, tracking the orientation of the attentional spotlight in audition is challenging. Kayser et al. (2005) have used a paradigm where they ask subjects to compare which of the two presented sound clips sounds more salient. Kim et al. (2014) let subjects listen to recordings of a conference room setting and indicate locations where they “hear any sound which you unintentionally pay attention to or which attracts your attention,” further defining salient locations as the ones that were indicated by nearly all subjects. Both studies compare the human experiment results with their computational models, but neither tackles the problem of quantifying the effect of specific auditory features or their interaction on saliency. Here, we follow a similar experimental approach by probing stimulus-related attentional perception using single sound clips, and asking listeners whether they heard a salient event. This paradigm allows us to construct structured full-factorial experiments that can map interactions between features with high statistical power. Although this paradigm is not free from top-down effects on attention, it has been argued that it can successfully account for bottom-up attention effects (Borji et al., 2013b).

The current work is guided by the hypothesis that as sounds evolve in a multi-dimensional feature space, regularities among features are tracked, and deviations from these regularities are “flagged” as salient. A broad range of natural stimuli is used to shed light on the conspicuity of and interactions between the dimensions of pitch, timbre, intensity, and timing in busy acoustic scenes. These perceptual features encapsulate much of the information that is extracted from the cochlea to mid-brain (Yang et al., 1992). A limited number of studies have established the existence of two-way interactions in the perception of some of these features (Melara and Marks, 1990; Allen and Oxenham, 2013); however, the extent of these interactions pertaining to attention is yet unknown. Here, we probe the effect of these features on auditory attention in a series of full-factorial pyschoacoustical experiments, in an attempt to map the entire interaction space. The same paradigm is used in each experiment, with different modalities of stimuli (musical tones, bird sounds, speech). Short sound clips containing temporally overlapping tokens of sound (e.g., musical note, word) varying in a small range of feature parameters form the scene's “background.” Only one token in the scene, the “foreground,” is manipulated according to factorial conditions to have a larger feature difference than the background tokens, and could appear at any moment in the scene. Upon presentation of a scene, the subject reports whether they heard a salient event. Results of the behavioral experiments demonstrate the principles governing the influence of acoustic properties on stimulus-induced attention.

In line with our stated hypothesis, we develop a computational model providing an implementation of predictive-coding to test for the first time whether the Bayesian brain framework can explain the perception of auditory saliency revealed by our behavioral experiments. The model analyzes the evolution of sound attributes over time, makes predictions about future values of sound features based on past regularities, and non-linearly integrates any flagged deviances to yield a unified estimate of saliency over time. The output of this computational model is contrasted with the pyschoacoustical findings from the behavioral experiments, providing a springboard for exploring the role of inference, predictive representations, and non-linear sensory interactions in mediating attention in audition.

## 2. Methods

### 2.1. Experiments

**Figure 1:**
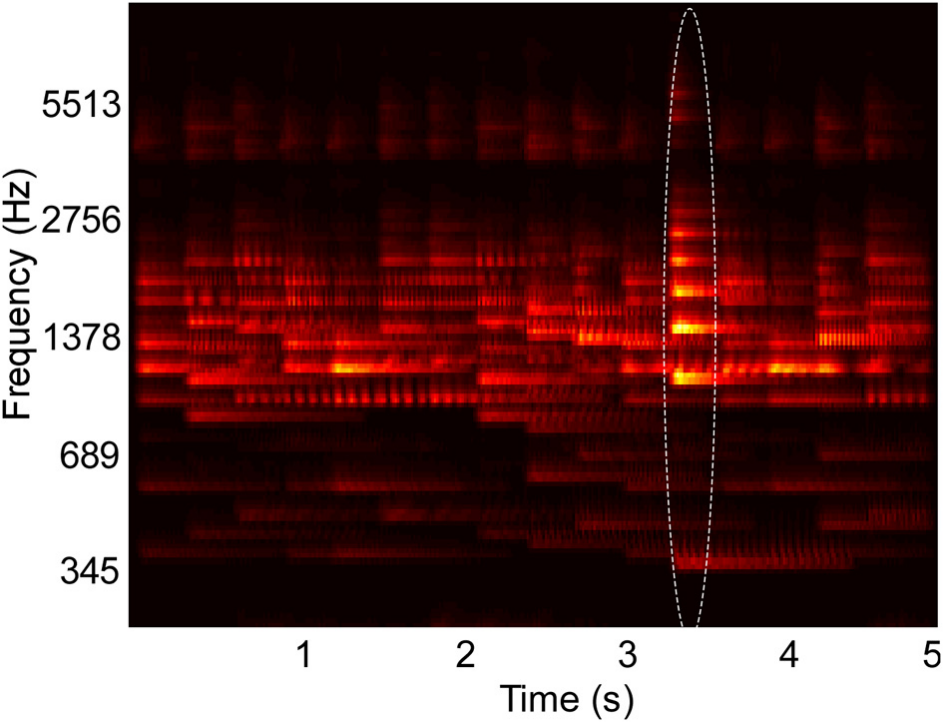
**Example spectrogram of stimulus used in behavioral experiments**. The spectrogram shows overlapping musical note tokens that compose a scene's background, and one foreground note, outlined in the image. Their pitch and intensity values are sampled from a constrained distribution of values, emulating a busy scene with natural sounds (Background pitch between 196 and 247 Hz). Listeners cannot perceive any individual note but are able to tell the class of sounds playing in the background. One “foreground” note that varies in pitch (Foreground pitch at 350 Hz) and intensity (6 dB higher than background notes) is introduced at a random location in the scene. In Experiments I and II, foreground tokens only appear in the second half of the scene, while in Experiment III, they can occur at any time. In all experiments, foreground tokens differ from the background in one or more of the following features: Pitch, intensity, and timbre. In the example shown in the figure, timbre was not varied. All tokens were clavichord notes.

Healthy subjects with normal hearing participated in the experiments with informed consent, as approved by the institutional review board at the Johns Hopkins University, and were compensated for participation. Subjects were Johns Hopkins University students and scholars with an average age of 22.6 (number of subjects were Exp. I: 13, Exp. II: 10, Exp. III: 10). All experiments have the same set-up: Subjects listen to short sound clips through Sennheiser HD595 headphones in a sound proof booth and answer saliency-related questions on a computer. All subjects in a given experiment listen to the same trials in randomized order. Each trial is presented only once. Trials consist of a dynamic background constructed by many sound tokens that overlap in time with varying density depending on the experiment (Figure 1). Background tokens are randomly selected from a pool of suitable tokens, leading to unique overall backgrounds in each trial. Backgrounds are manipulated so that there is a uniform distribution of frequencies over time, to minimize coincidental increases in pitch difference between the background and foreground tokens. Control trials consist of just the background scene, while test trials have one “foreground” salient token in addition to the background. The foreground token differs from background tokens in one or more of the experiment factors (i.e., acoustic attributes of the foreground token). Following each trial, subjects are asked “Does the clip contain a salient event?” and report Yes/No answers without feedback. Each experiment is preceded with a brief training session comprised of 7–12 trials that are similar to experimental trials but with feedback provided about which sound feature is changed in the foreground token. Subjects can adjust sound intensity to their individual comfort level in all experiments, at any time during the experiment.

Subject performance is measured with the *d*′ metric, which accounts for false detection rate along with the correct detection rate. In the calculation of *d*′, the detection rate changes according to factorial conditions (averaged between the repetitions of the factorial condition), however, the false detection rate is constant for each subject (average of all control trials for the duration of the experiment, since there is no way to attribute a false detection to a particular factor). For both correct and false detection rates, values of 0 and 1 are adjusted to 0.01 and 0.99, respectively. This adjustment is in line with corrections commonly used for *d*′ measures to avoid infinite values. It is worth noting that similar results are obtained irrespective of the small adjustments to the correct and false detection rates. In the analysis of each experiment, the *d*′ was calculated for each factorial condition for every subject. All performed ANOVAs are fully within subjects, where every feature is treated as a fixed effect, and individual error terms are used in the calculation of the *F* statistic. The Benjamini-Hochberg procedure (Benjamini and Hochberg, 1995) is used to iteratively validate the significance levels for multiple comparisons shown in Tables 1, 2.

**Table 1:** **ANOVA results of human experiments**.

**Effects**	**F (p)**		
	**Music**	**Nature**	**Speech**
**Pitch**	**17.76 (<0.01)**	**211.69 (<0.01)**	**103.76 (<0.01)**
**Intensity**	**14.08 (<0.01)**	**17.57 (<0.01)**	**98.50 (<0.01)**
Timbre-bg	0.63 (0.54)	**8.66 (<0.01)**	**71.21 (<0.01)**
Timbre-fg	2.11 (0.14)	**52.51 (<0.01)**	**29.12 (<0.01)**
**P, I**	**7.36 (0.02)**	**18.00 (<0.01)**	**134.58 (<0.01)**
P, T_b_	0.51 (0.61)	0.09 (0.91)	**19.13 (<0.01)**
P, T_f_	1.77 (0.19)	**36.21 (<0.01)**	**12.19 (<0.01)**
I, T_b_	1.09 (0.35)	0.98 (0.39)	0.03 (0.86)
I, T_f_	0.13 (0.88)	**9.72 (<0.01)**	**11.40 (<0.01)**
**T_b_, T_f_**	**13.29 (<0.01)**	**30.21 (<0.01)**	**13.22 (<0.01)**
P, I, T_b_	0.28 (0.76)	3.06 (0.07)	**7.03 (0.03)**
P, I, T_f_	1.23 (0.31)	0.60 (0.56)	0.39 (0.55)
**P, T_b_, T_f_**	**6.77 (<0.01)**	**36.85 (<0.01)**	**33.21 (<0.01)**
I, T_b_, T_f_	1.57 (0.20)	0.18 (0.95)	**5.60 (0.04)**
P, I, T_b_, T_f_	0.29 (0.90)	0.24 (0.91)	**7.47 (0.02)**

**Table 2:** **ANOVA results of interactions including the Time factor in the Experiment III**.

	**F (p)**		**F (p)**
**Time**	**42.57 (<0.01)**	Time, I, T_b_	2.57 (0.08)
**Time, P**	**18.90 (<0.01)**	Time, I, T_f_	1.76 (0.18)
Time, I	1.12 (0.32)	Time, T_b_, T_f_	2.77 (0.06)
Time, T_b_	2.17 (0.12)	Time, P, I, T_b_	2.06 (0.13)
Time, T_f_	1.61 (0.21)	Time, P, I, T_f_	0.56 (0.64)
Time, P, I	0.87 (0.47)	Time, P, T_b_, T_f_	0.15 (0.93)
Time, P, T_b_	1.43 (0.26)	Time, I, T_b_, T_f_	0.80 (0.51)
**Time, P, T_f_**	**4.75 (<0.01)**	Time, P, I, T_b_, T_f_	1.32 (0.29)

**Figure 2:**
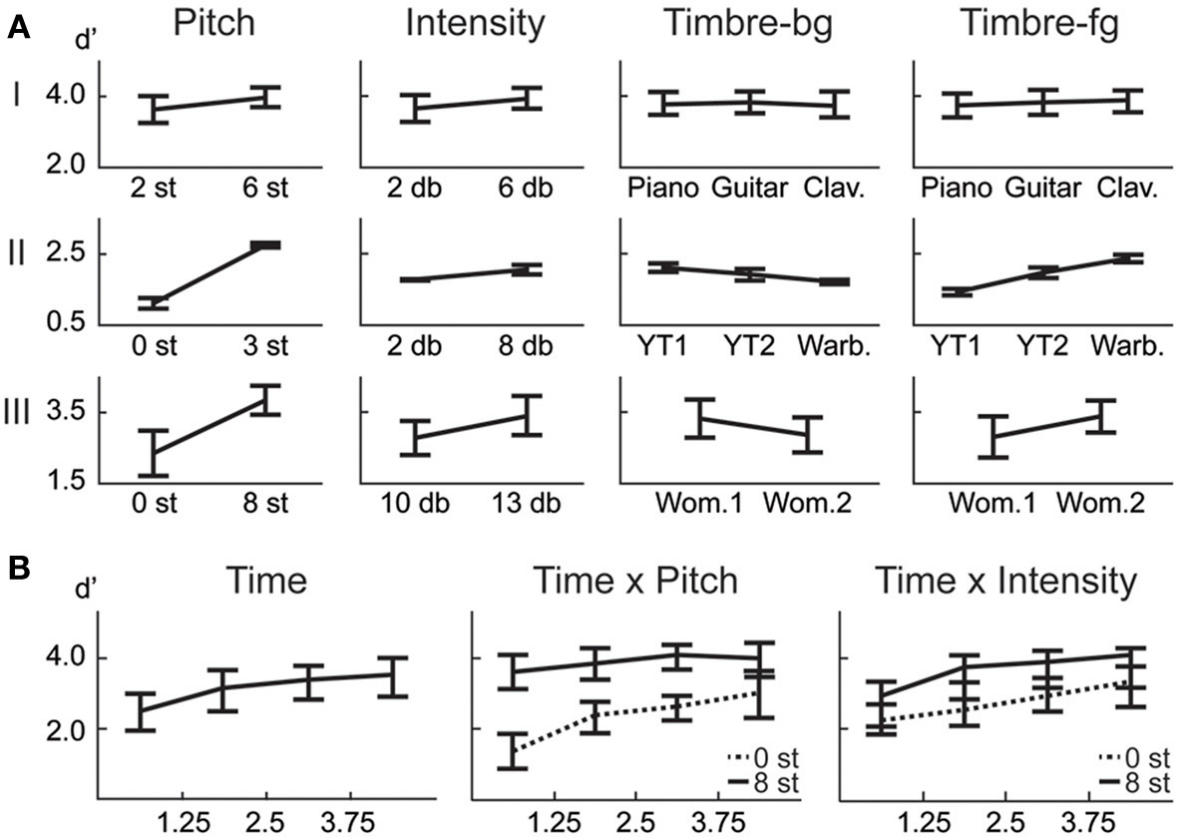
**Behavioral results. (A)** ANOVA main effect trends for all experiments. **(B)** The effect of the time factor reveals a temporal build-up observed in human detection of saliency. Interaction of time with pitch and intensity are shown. The significance levels corresponding to these plots can be found in Table 2.

Although the backgrounds in the trials are not identical, there is a possibility that subjects learn the backgrounds over time because of the limited set of background tokens. It is difficult to obtain speech and bird song data from the same source that have near identical pitches but are unique vocalizations. In the case of music, the number of musical notes is predetermined for each instrument, leading to a limited set of notes constrained in a small range of pitch. However, we examine the difference between number of errors in the first half vs. second half of each experiment, and find no significant difference (1-way within subjects ANOVA: Exp. I: *F* = 1.44, *p* = 0.24; Exp. II: *F* = 0.49, *p* = 0.49; Exp. III: *F* = 0.23, *p* = 0.64). Furthermore, results from Exp. III confirm that detection of tokens in the beginning of each trial is low throughout the experiment (Figure 2B), refuting the possibility of meta-learning.

#### 2.1.1. Experiment I: music

The first experiment uses a background of non-melodic natural instrument sounds. Non-sustained single notes from the RWC Musical Instrument Sound Database (Goto et al., 2003) are extracted for Pianoforte (Normal, Mezzo), Acoustic Guitar (Al Aire, Mezzo), Clavichord (Normal, Forte) at 44.1 kHz. Background notes range between 196 and 247 Hz (G3-B3). Each token is 1.2 s in duration and amplitude normalized relative to its maximum with 0.1 s onset and offset sinusoidal ramps. Four sequences of consecutive tokens, randomly chosen for each trial, are combined with 0.3 s phase delay to form a 5 s dynamic background. Each test trial has one foreground note at 2 or 6 semitones (278Hz-C#4, 350Hz-F4) and 2 or 6 dB higher than background, added at a randomly chosen onset time between 55% and 75% of the trial length. The resulting experiment design is (Pitch * Intensity * Timbre-foreground * Timbre-background) 2 * 2 * 3 * 3. Each test condition is repeated eight times (with non-identical backgrounds). 25% of trials are control trials. Control trial tokens vary in the same range of pitch and intensity as background tokens of test trials. One third of control trials use Pianoforte, one third Acoustic Guitar, and one third Clavichord.

The instruments in this experiment were manually selected such that the they are sufficiently distinguishable from each other, but not so much that listeners with normal hearing and musical training would detect each different note, as determined by short pilot investigations with few listeners. The difference levels for pitch and intensity were similarly set manually to result in a difference that can be definitely heard if one listens for it, but might be missed if not paying attention. The factor levels for subsequent experiments were also set with these criteria.

*Experiment I-2* An additional experiment is performed to validate the main effects of musical instruments on the perception of saliency. In this experiment, pitch (5 and 10 semitones higher and lower than the background mean), intensity (7 and 10 dB higher than the background tokens), and timbre are tested separately. Sustained single notes from the RWC Musical Instrument Sound Database (Goto et al., 2003) are extracted for Harmonica, Violin, Flute (Normal, Mezzo for each) at 44.1 kHz, and downsampled to 16 kHz. Background notes range between 587 and 740 Hz (D5-F#5). Each token is 1 s in duration and amplitude normalized relative to its top 10%th value with 0.5 s onset and 0.01 s offset sinusoidal ramps. Tokens overlap every 0.5 s, forming two sequences. The foreground token varies in only one of the dimensions with respect to the background, and is placed at a random onset between 50% and 80% of the trial length. In each trial, subjects are presented two sound clips, one or none of which contains a salient token. The subject is asked “Which clip contains a more salient event?” and is presented the options “Clip 1”/“Clip 2”/“Equal.” Each condition is repeated four times, with additional 20% control trials.

#### 2.1.2. Experiment II: nature

The scene setup of this experiment is a busy natural forest environment with singing birds. Natural song recordings of two different Common Yellowthroats, and one MacGillivray Warbler are obtained from the Macaulay Library (http://macaulaylibrary.org, reference numbers: 118601, 136169, 42249). Individual calls at approximately 4.9 kHz pitch and 1.3–1.5 s length are manually extracted at 44.1 kHz. Recordings of wind and water sounds are added to every trial to reduce signal-to-noise ratio, and make the task more challenging while retaining the “natural” scene set-up. Due to unavailability of higher pitched calls from the same bird, background tokens are manually shifted three semitones higher with Adobe Audition to be used as foreground tokens. Additional foreground songs with 0 semitone pitch difference are also used, with a change in another attribute (intensity or timbre) following the factorial experimental design. Tokens are amplitude normalized relative to their top 5%th value. Recordings of water and wind sounds (one track for each) are each normalized to have the same peak amplitude as the combined background, and further added to the background. The foreground token is 2 or 8 dB higher than the background. Three sequences of bird calls with 0.5 s phase shift are added for a total duration of 6 s. The foreground token onset is randomly chosen between 58% and 68% of the trial length. Each individual background token is used at most two times within the same trial. The resulting experiment design is (Pitch * Intensity * Timbre-foreground * Timbre-background) 2 * 2 * 3 * 3. Each condition is repeated eight times with additional 25% control trials. Control trial tokens vary in the same range of pitch and intensity as background tokens of test trials. Each third of the control trials uses one of the three bird sounds in this experiment.

#### 2.1.3. Experiment III: speech

The background in the third experiment emulates a party scene where one can perceive that people are speaking, but cannot make out what is being said. A noisy telephone conversation recording of two female Japanese speakers is selected from the CALLHOME Database (http://www.ldc.upenn.edu/Catalog/CatalogEntry.jsp?catalogId=LDC96S37). The choice of Japanese in this experiment is deliberate to ensure non-linguistic interpretations from our non-Japanese-speaking listeners. Further, unlike in Exp. I, one cannot make out individual tokens even while actively attending to them, due to the high level of word overlap and noise in the source recording. Fifty-six words in the 175–233 Hz (F3-A#3) range and of 0.5–1.2 s length are manually extracted at 8 kHz to be in the background. Each word is allowed to appear at most twice in one trial. Each token is amplitude normalized with its top value and applied a 0.05 s long onset and offset ramp. The background consists of a combination of four sequences of tokens with no delay. Foreground tokens are 10 and 13 dB higher from the cumulative background. A foreground token consists of a sample from a selection of 12 words with approximately eight semitone difference from the background between 349 and 369 Hz (F4-F#4), each 0.5 s long. Additional foreground words with 0 semitone pitch difference are also used. The foreground onset is also manipulated by placing it in one of four 1.25 s long quadrants of the 5 s long trial, hence probing the effect of timing of foreground on perception of saliency. The resulting experiment design is (Pitch * Intensity * Timbre-foreground * Timbre-background * Time) 2 * 2 * 2 * 2 * 4. Each condition is repeated four times, 7.25% are control trials. Control trial tokens vary in the same range of pitch and intensity as background tokens of test trials. Sixty percent of control trials use one speaker, while forty percent use the other speaker.

### 2.2. Computational model

#### 2.2.1. Computation of sound features

**Figure 3:**
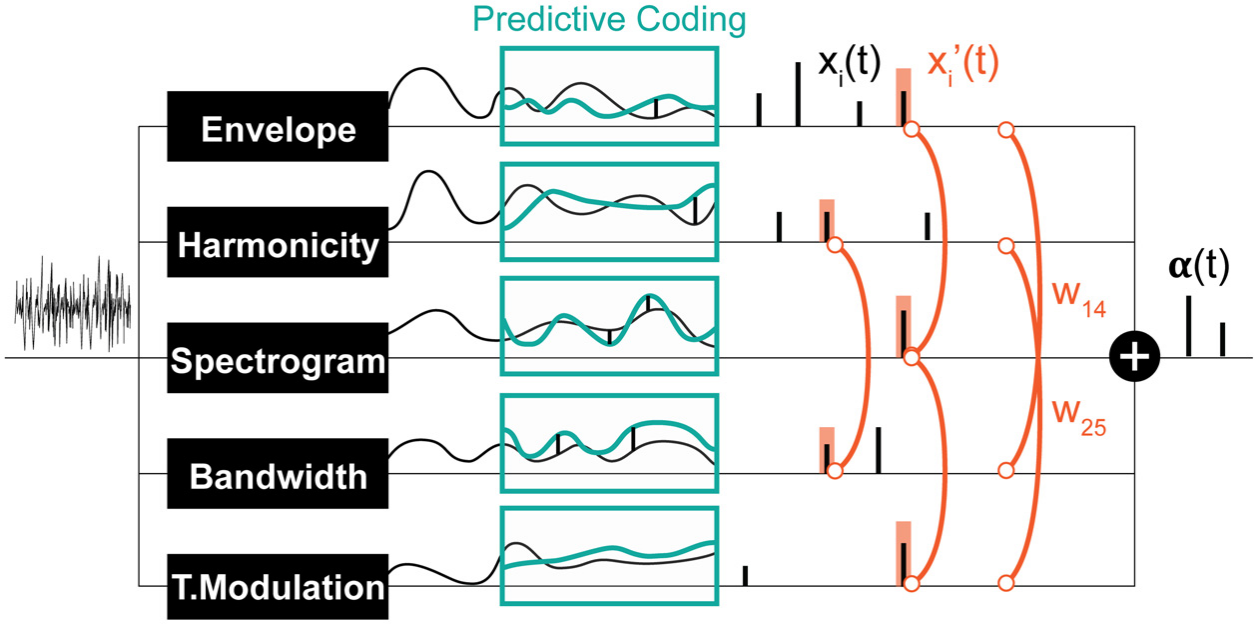
**Schematic of the computational saliency model**. The model is structured along three stages. It starts with an acoustic waveform and extracts relevant features along five dimensions. Regularities within each feature dimension are then tracked used a Kalman-filter to make predictive inferences about deviations from ongoing statistics in that corresponding feature. Detected deviants are boosted according to interaction weights learned using the experimental stimuli, then integrated across feature dimensions to yield an overall saliency estimate of the entire auditory scene. The final values mark salient timings in the scene.

The model starts by extracting acoustic attributes of the incoming signal with a focus on intensity, pitch and timbre (Figure 3). Intensity is derived from an estimate of the signal's temporal envelope, extracted from the magnitude Hilbert transform, Butterworth filtered with *w_c_* = 60 Hz, *n* = 6. Pitch and timbre are extracted from the sound spectrogram, which is computed with 1 ms frames. The spectrogram computation mimics the processing known to occur from the cochlea to the mid-brain: Using a bank of 128 constant-Q bandpass log-scale filters, followed by high-pass, compression, and low-pass filtering then spectral sharpening following the model of Chi et al. (2005). Pitch is extracted from a harmonicity analysis of spectrogram spectral slices, following a template matching approach (Shamma and Klein, 2000; Walker et al., 2011). Only pitch estimates with a good match to the template are retained, and further smoothed using a median filter with a 5-sample window. Timbre is a more abstract, less quantifiable attribute, than pitch or intensity. Earlier work argued a close correspondence between timbre perception and spectro-temporal details of sound events (Patil et al., 2012). Here, we follow the same premise and first augment our feature space directly with the channels of the spectrogram. In addition, we extract bandwidth information that highlights broad vs. narrowband spectral components; along with temporal modulations that follow dynamic changes of sounds over time. The temporal response of each spectrogram channel is analyzed using short-term Fourier transform with 200 ms windows with 1 ms overlap. Spectral slices of the spectrogram are processed further using Gabor bandpass filters with characteristic frequencies logarithmically distributed between 2^−2^ and 2^4^ cycles/octave to extract bandwidth details (Chi et al., 2005). The top 64 and bottom 64 channels of the spectrogram are treated as separate features in subsequent processing as high and low frequency spectrum features. The full mapping consists of a 167-dimensional tensor. Finally, each computed feature is further binned using 200 ms windows, such that the mean of the window is assigned to every sample in the window.

#### 2.2.2. Deviance detection on feature streams

Following the framework of predictive-coding, each of the model features (envelope, harmonicity, and each frequency channel in high-frequency spectrogram, low-frequency spectrogram, bandwidth, temporal modulation) is separately tracked over time by a Kalman filter (Chen, 2003), which is a linear dynamical system that estimates the channel's state based on measurements over time, by minimizing the least square error between the predicted and observed input. The Kalman filter is used because it is efficient, versatile, and simple to implement and interpret. At each feature channel, clustering on a short segment at the start of the feature decides the regularities to be predicted for that feature. Each regularity stream is tracked with a separate Kalman filter, leading to multiple predictions for incoming values among each feature. If a feature does not fit any of the Kalman predictions, it produces a spike at that instant, signaling a deviant; and a Kalman filter for this novel value is initialized. Filters that are not updated for one second are reset. The match between the input and prediction is determined by a dynamic threshold that depends on prior prediction accuracy. Consequently, if predictions have been matching the input for some time, the expectation is that predicted values will keep being encountered, leading to a decrease in the fit threshold. As the dynamical system evolves, a series of spikes are generated corresponding to times of salient events. The amplitude of each spike corresponds to the difference between the real feature measurement at that time and the closest prediction window. Finally, spike trains from multi-channel axes (e.g., different frequency channels in the high-frequency spectrogram) are grouped together. If there are multiple spikes at the same time instant, the maximum one is recorded.

The underlying linear system for the Kalman filters in our model is:

$$\begin{array}{l} A(t)=FA(t-1)+u(t)\\ Z(t)=HA(t)+v(t) \end{array}$$

where *A* is the time-dependent state (or feature variable) being tracked. *Z* is the observed input. *u* and *v* are small Gaussian noise perturbations, modeled respectively as:


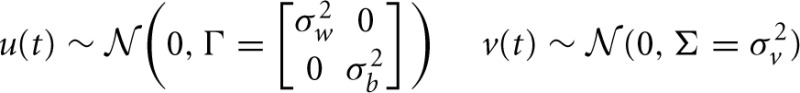


The variances of the noise parameters are empirically chosen for each feature; set to σ_*w*_ = 0.001, σ_*b*_ = 0.01, and σ_*v*_ = 0.06 for envelope and pitch, σ_*w*_ = 0.00025, σ_*b*_ = 0.0025, and σ_*v*_ = 0.0125 for spectrogram, bandwidth, and temporal modulation. The state vector and the system matrices reflect a random walk, and can be encoded as:

$$A(t)\;=\left[\begin{array}{c} Z(t)\\ Z(t)-Z(t-1) \end{array}\right]\;F\;=\left[\begin{array}{cc} 1 & 1\\ 0 & 1 \end{array}\right]\;H\;=\left[\begin{array}{cc} 1 & 0 \end{array}\right]$$

The number of regularity streams (each represented with a separate Kalman) to initialize for each feature is determined by k-means clustering of the first 125 ms of feature values. The numbers of clusters are selected so that the sum of distances within each cluster is smallest. For each of these clusters, a Kalman filter is initialized as shown below. The initial values for the state prediction error are calculated from the last two sample values of the initialization window: If *n*_*i*_ denotes the sample number at 125 ms, the initial estimate for the state vector, and its corresponding state prediction error covariance then becomes:

$$\begin{array}{l} \;\hat{A}(t)=\left[\begin{array}{c} 2Z(n_{i})-Z(n_{i}-1)\\ Z(n_{i})-Z(n_{i}-1) \end{array}\right]\\ \hat{\Psi }(t)\;=\left[\begin{array}{cc} 5\sigma_{v}^{2}+2\sigma_{w}^{2}+\sigma_{b}^{2} & \sigma_{w}^{2}+3\sigma_{v}^{2}+\sigma_{b}^{2}\\ \sigma_{w}^{2}+3\sigma_{v}^{2}+\sigma_{b}^{2} & 2\sigma_{v}^{2}+\sigma_{w}^{2}+2\sigma_{b}^{2} \end{array}\right] \end{array}$$

Next, at every time instance, the model iteratively computes its Kalman gain *K*(*t*), and updates its posterior estimate of the state *Â*(*t*) and $\hat{\Psi }$(*t*); following the equations:

$$\begin{array}{l} \;K(t)=(F\hat{\Psi }(t-1)F^{T}\text{​}+\Gamma )H^{T}(H(F\hat{\Psi }(t-1)F^{T}\text{​}+\Gamma )H^{T}\text{​}+\Sigma )-1\\ \;\hat{A}(t)=F\hat{A}(t-1)+K(t)(Z(t)-HF\hat{A}(t-1))\\ \hat{\Psi }(t)\;=(I-K(t)H)(F\hat{\Psi }(t-1)F^{T}+\Gamma ) \end{array}$$

The threshold to determine whether an input value fits into the prediction of a Kalman is an adaptation from (Arnaud et al., 2005):

$$|Z(t)-HF\hat{A}(t)|\;\leq \sqrt{4({\hat{\Psi }}_{\left[1\right]}+\sigma_{v}^{2})}$$

where $\hat{\Psi }$_[1]_ is the first element in the matrix $\hat{\Psi }$.

#### 2.2.3. Integration of saliency information among features

The result of Kalman filtering is a set of one dimensional spike signals for each feature, shown in Figure 3 as *x_i_(t)*, where *t* is time, and *i* ∈ [1, *n*] (*n* = 6 in our case). These spikes represent some probability of having a salient event at the time instance in which they occurred; the higher the value, the more likely is saliency. Note that spike amplitudes in each signal reflect relative deviance within that feature and are not globally normalized to values in other signals. We normalize contribution of each feature and non-linearly model integration interactions with constrained logistic regression, using the stimuli used in our experimental paradigm with their corresponding ground truth about the timings of salient sounds (i.e., timing of foreground tokens).

Let *y(t)* be a binary variable representing the existence of a salient event in time *t*. Our objective is to learn a mapping from *x_i_(t)* ∈ [0, ∞] to *P(y(t)* = 1) ∈ [0, 1]. An intermediate step in this mapping is boosting the signals (resulting in *x*′_*i*_(*t*)) with asymmetric interaction weights between feature pairs. This process is illustrated in Figure 3 and modeled as:

$${x^{\prime }}_{i}(t)=x_{i}(t)(w_{ii}+\sum_{\begin{array}{c} j\;\in \;[1,n]\\ j\;\neq \;i \end{array}}w_{ij}\underset{{}_{k\in [-s,s]}}{\max}x_{j}(t+k))$$

*w_ij_* are the asymmetric interaction weights between feature *i* and feature *j* that we want to find the optimal values of. The window *s* around a spike accounts for timing shifts due to sampling and is set here to 7 ms. This process is illustrated in Figure 3. The optimal weights *w*_*ij*_ are computed using experimental stimuli. The ground truth about deviants in each channel *i* in these stimuli is:

$$y_{i}(t)=\{\begin{array}{ll} 1, & \text{for }t\text{ within salient event duration}\\ 0, & \text{otherwise} \end{array}$$

We use constrained logistic regression (MATLAB Optimization Toolbox) to map between *x*′_*i*_(*t*) and *y*_*i*_(*t*). The probability of having a salient event in feature *i* at time *t* is determined by:

$$\alpha_{i}(t)=p(y_{i}(t)=1)=\frac{2}{1+e^{-{x^{\prime }}_{i}(t)}}-1$$

and the corresponding probability of not having a salient event is:

$$p(y_{i}(t)=0)=1-p(y_{i}(t)=1)=\frac{2e^{-{x^{\prime }}_{i}(t)}}{1+e^{-{x^{\prime }}_{i}(t)}}$$

With the given binary definition of *y*_*i*_(*t*), the probabilities above can be written concisely as:

$$p(y_{i}(t)|{x^{\prime }}_{i}(t))=\frac{y_{i}(t)+{\left(-1\right)}^{y_{i}(t)}2^{\left(1-y_{i}(t)\right)}e^{-{x^{\prime }}_{i}(t)}}{1+e^{-{x^{\prime }}_{i}(t)}}$$

leading to the log-likelihood function:

$$\underset{w_{ij}}{\max}\sum_{t}log(\frac{y_{i}(t)+{\left(-1\right)}^{y_{i}(t)}2^{\left(1-y_{i}(t)\right)}e^{-{x^{\prime }}_{i}(t)}}{1+e^{-{x^{\prime }}_{i}(t)}})\text{ st. }w_{ij}\geq 0$$

Due to the positive constraint on the weights, *x*′_*i*_(*t*) is also constrained to be positive, hence limiting the regression to only the positive part of the logistic function. The optimization is performed simultaneously on all features; with clips from all experiments (and their correspondent ground truths) incorporated as training data. For analyses where each experiment is trained separately, each feature is also optimized separately to reduce noise. With the learned weights plugged in, the final output of the entire model is α(*t*), the likelihood of saliency among time, a value in [0, 1].

## 3. Results

### 3.1. Experiments

#### 3.1.1. Experiment I: music

In this first experiment, we investigate the effect of pitch, intensity, and timbre on perception of saliency. Because timbre is a non-numeric attribute, we probe the effect of each musical instrument as a foreground (T_f_) and background (T_b_) timbre event. Pitch (P) and intensity (I) are found to have significant effects (Table 1). However, neither background nor foreground timbre factors have significant effects. Marginal means (Figure 2A) confirm that the three instruments are indeed relatively close to each other in timbre space; as corroborated by published studies of timbre perception (McAdams et al., 1995). A follow-up study (Exp. I-2) reveals that the lack of timbre effect is specific to the choice of instruments. An experiment with violin, harmonica and flute [instruments with a wider timbre span (McAdams et al., 1995)] shows a statistically significant saliency effect of both foreground and background timbres (*F_P_* = 4.23 *p_P_* = 0.046, *F_I_* = 16.44 *p_I_* < 10^−2^, *F_T_b__* = 8.31 *p_T_b__* < 10^−2^, *F_T_f__* = 4.00 *p_T_f__* = 0.02).

#### 3.1.2. Experiment II: nature

Overall, this natural sound experiment is more difficult than the musical notes task (overall *d*′: 1.88 compared to 3.61); but reveals that all four factors have significant effects (Table 1). The consistency of effects between Exp. I and II argues against possible ceiling confounds that could have resulted from the musical notes experiment.

#### 3.1.3. Experiment III: speech

In this experiment, we probe the effect of time in addition to the same three attributes tested earlier. Time refers to the placement of the foreground token in the scene, appearing in four possible time-quadrants. All tested factors are found to influence saliency (Figure 2). The trend of the time factor implies that the later a deviant sound is heard in a scene, the more salient it is perceived. There is a significant *d*′ increase in the first two quadrants of the scene (Bootstrap 95% confidence interval for slope: (25.6°, 35.8°), *p* < 10^−2^), indicating rapid adaptation to the background (Figure 2B). The trend stabilizes later in time (low difference between last two quadrants; Bootstrap 95% confidence interval for slope: (−1.1°, 16.7°), *p* = 0.09) implying that once standard formation has taken place, detection may no longer be highly dependent on exact timing.

#### 3.1.4. Interactions

**Figure 4:**
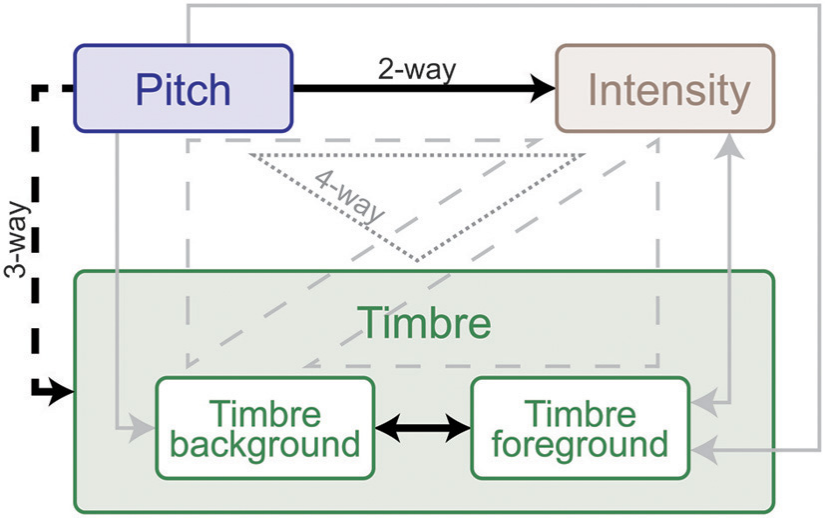
**Summary of interaction weights based on behavioral tests with human listeners**. Solid lines indicate two-way, dashed lines three-way and dotted lines four-way interactions. Effects that emerged in every experiment are shown black, and those that were found in at least one experiment are shown gray. Arrow directions indicate direction of interaction: the origin feature has a relatively larger effect on the destination feature in all experiments. Double-sided arrows indicate that there is no clear weight either way. The weight and directionality of interactions observed are inferred from the coefficients of the fitted model, and are limited by the levels of sound features tested in this study.

An interaction between multiple factors indicates that the effect of one factor changes according to the levels of the others. Within-subjects ANOVA results, outlining the interactions from all experiments, are shown in Table 1. Intensity and pitch have a significant interaction: The effect of intensity is more prominent when pitch difference is low. Although separate timbre components (T_f_, T_b_) are not significant in every experiment, their interaction is significant; demonstrating that the effect of timbre on saliency stems from the interplay of background and foreground. Further, while T_f_ and T_b_ do not separately interact with pitch in every experiment, the combined interaction PxT_b_xT_f_ does. Thus, one can argue that pitch and timbre have a significant interaction (Figure 4). An interaction between intensity and timbre, and between all four factors, is observed in only one experiment.

Time emerges as an additional significant factor in Exp. III. In one case, the effect of pitch on perceived saliency is found to depend on the length of build-up (Figure 2B). The complete high-level interactions can be found in Table 2, corroborating the importance of timing of events for auditory saliency. The higher detection performance when the salient event is later in the scene suggests a notion of accumulation of background statistics over time, in agreement with our hypothesis.

### 3.2. Computational model

**Figure 5:**
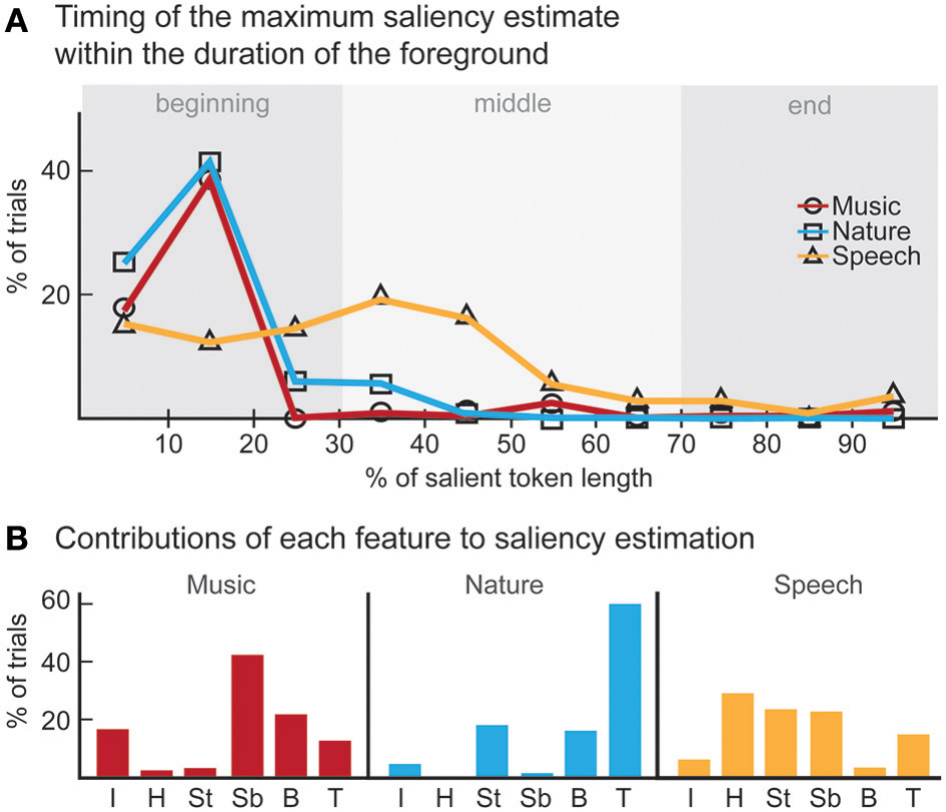
**Analysis of model results. (A)** The time instance where the maximum likelihood of saliency was detected for foreground tokens in the scene. Trials in which the maximum saliency was found outside the duration of the foreground are not included. For musical notes and bird songs, the deviance is detected soon after the token onset. For spoken words, the deviance is detected during the first half of the token onset. In some cases, the model finds the offset deviance instead of onset deviance. **(B)** Regardless of whether the maximum likelihood of saliency was inside the foreground token duration, the feature that the saliency was detected in is shown. The features are, in order: Envelope, Harmonicity, Spectrogram-top, Spectrogram-bottom, Bandwidth, Temporal modulation.

**Figure 6:**
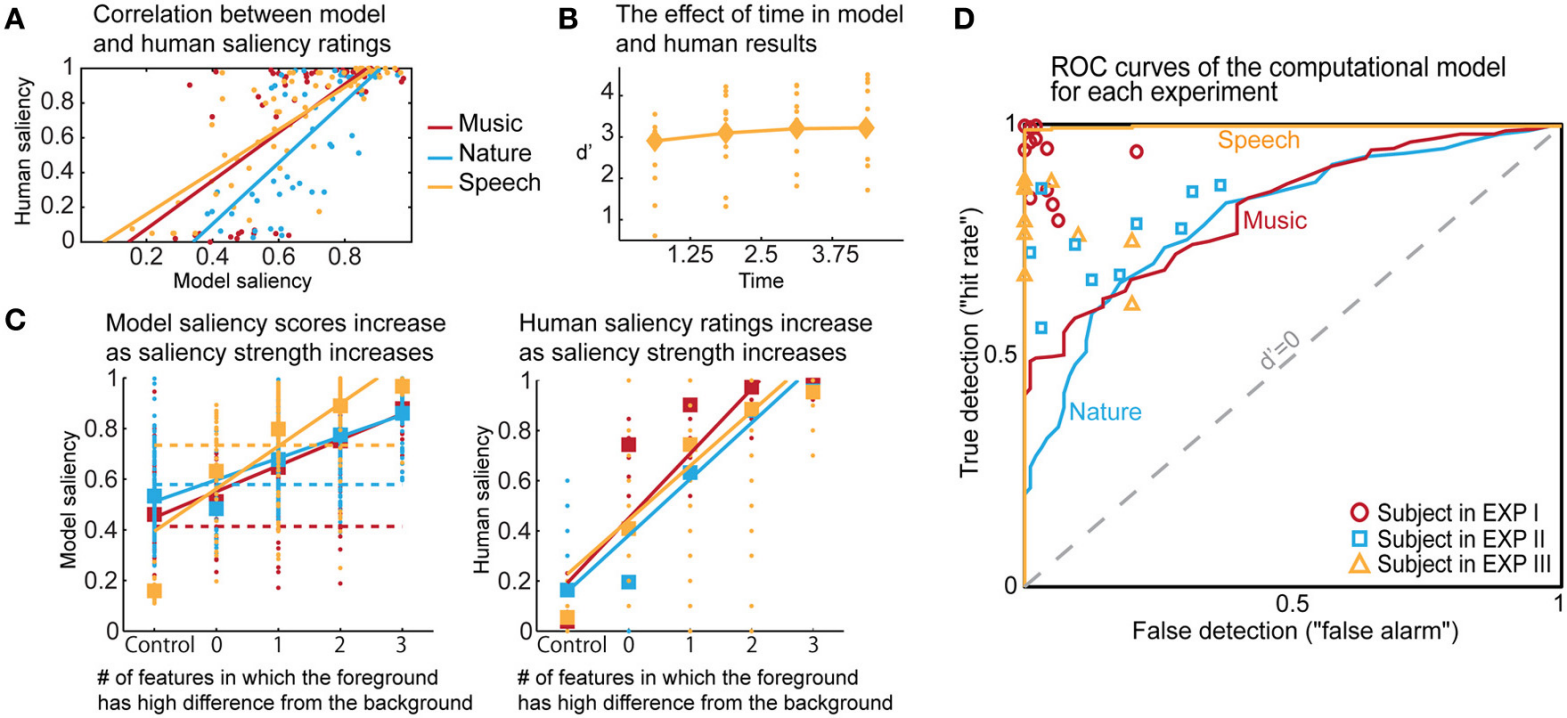
**Comparisons of human and model results based on saliency ratings and detection performance. (A)** Correlation between averaged model saliency scores and human saliency ratings shown for all experiments. Averaging is performed between repeated experimental cases, and also between subjects for the human ratings. **(B)** The time trend that emerged in the model results for Experiment III. Diamonds show the *d*′ for each quadrant in model results, and dots represent the human responses. We observe a similar trend as in Figure 2B. **(C)** We show that as saliency increases, the model produces higher saliency scores. This is along the same lines with human results. Control trials have no foreground token; there is no salient event during the scene. Feature level 0 on the *x*-axis corresponds to a foreground token with low level of saliency. As an example, for Experiment III, this corresponds to no difference in pitch or timbre, but a 10 db difference in intensity. Feature level 1 corresponds to the high level of difference, which is 13 db for intensity in this experiment. Any change in timbre or pitch is also counted as a high difference due to the experimental set-up, outlined in Methods. The dashed lines in the left plot show where the threshold falls for calculating the optimal *d*′. The separability of control trials from test trials demonstrated here is also reflected in the ROC plot. **(D)** The probabilistic output of the saliency model leads to a detection curve in ROC space by setting a threshold to distinguish true and false detections. The *d*′ metric can be computed for each point in this space, quantifying performance; *d*′ is 0 when true and false detection rates are equal. We can infer from the curves that the saliency scores of the control trials are most easily separable than the saliency scores of the test trials for Experiment III, and that the performance of the model is closest to humans for Experiment II.

The computational model produces a one-dimensional signal indicating the likelihood of salient events over time, corresponding to a “saliency score.” The model is run on the same stimuli used in the experiments, with interaction weights obtained by training on the ground truth about salient events. Note that no model training is done to match it to the human ratings. The average model saliency scores for trials with salient tokens are statistically significantly higher than those for control trials (*t*-test, all experiments: *p* < 10^−2^). In most trials, the likelihood of saliency is highest during the duration of the actual salient event: I: 61%, II: 78%, III: 92% (Figure 5A). When contrasting the model scores with human ratings, strong correlations are observed (Figure 6A). The saliency scores of repeated factorial cases are averaged for the model. The human responses, mapped to 0 and 1, are averaged over factorial case repetitions, and also averaged between subjects. Statistically significant correlations are found in each experiment, when the model weights are calibrated for stimuli and ground truth from all experiments simultaneously (Spearman's rank correlation: I: ρ = 0.60, *p* < 10^−5^. II: ρ = 0.63, *p* < 10^−5^. III: ρ = 0.61, *p* < 10^−5^.). Higher performance is observed when the model is calibrated for ground truth of each experiment separately (Spearman's rank correlation: I: ρ = 0.64, *p* < 10^−5^. II: ρ = 0.72, *p* < 10^−5^. III: ρ = 0.80, *p* < 10^−5^.). Furthermore, we observe that the model saliency scores increase as the level of saliency increases. The level or strength of saliency of a token is taken as the number of sound attributes in which the foreground is different than background. Figure 6C (left) shows the increase in model saliency score as the foreground saliency strength increases (Spearman's rank correlation: I: ρ = 0.67, *p* < 10^−5^, II: ρ = 0.61, *p* < 10^−5^, III: ρ = 0.64, *p* < 10^−5^). The behavior of human listeners is also similar, with average ratings across subjects increasing as strength of saliency increases as shown in the right plot in Figure 6C (Spearman's rank correlation: I: ρ = 0.83, *p* < 10^−5^, II: ρ = 0.81, *p* < 10^−5^, III: ρ = 0.64, *p* < 10^−5^).

**Figure 7:**
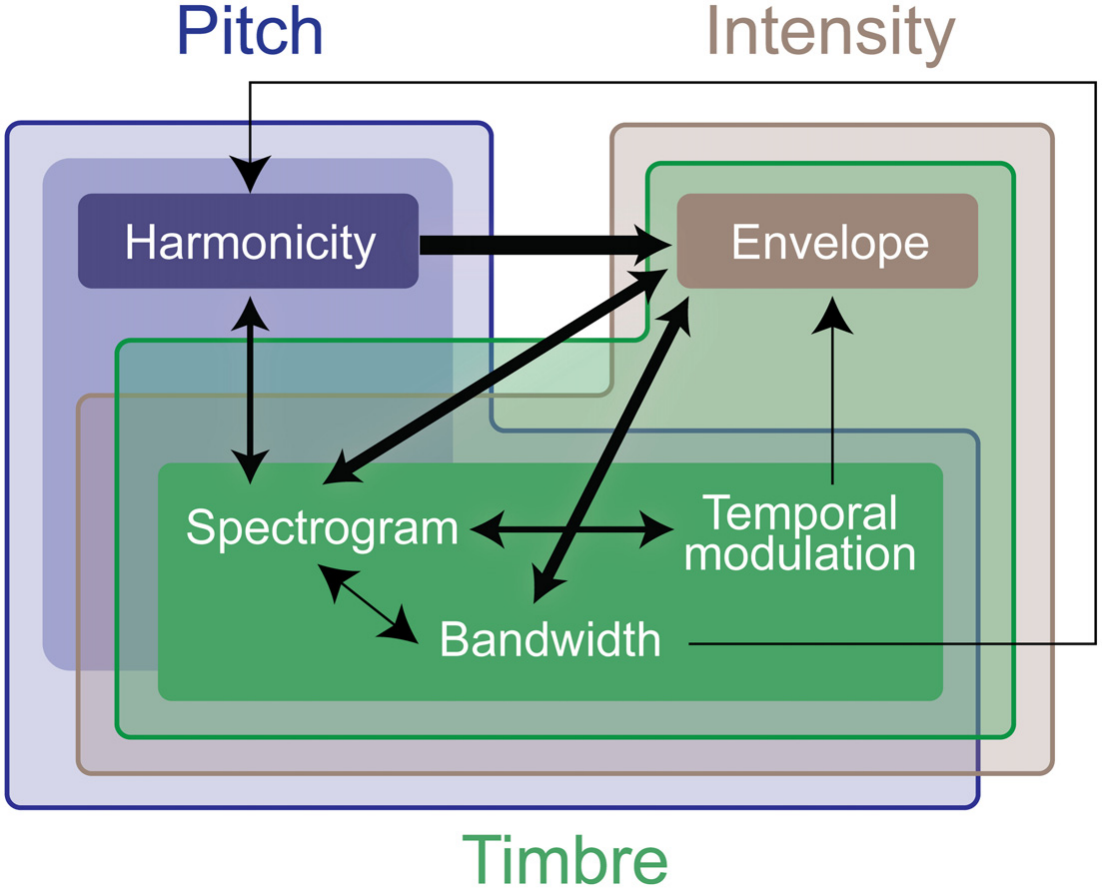
**Summary of interaction weights that emerge from training the computational model**. The model is trained using the same stimuli used in the experimental testing. Thicker lines denote higher weights. An arrow between features indicates that the origin feature of the line boosts the effect of the destination of the line. The different colors indicate the computational features that encode effects of the experimental features, the deeper the color, the stronger the relationship. As in Figure 4, the weight and directionality of interactions in this figure are inferred from the coefficients of the fitted model, and are limited by the levels of sound features tested in the human experiments.

We perform further analysis on the model's behavior and observe that different acoustic features have varying levels of contribution in different experiments; bandwidth and temporal modulation appear to be the most effective (Figure 5B). A careful inspection of model feature interactions shows strong similarity with psychoacoustic findings (Figures 4, 7), even though the model interaction weights are trained based on ground truth about deviant events, not on human results. In particular, pitch and intensity have a strong interaction in both human perception and the computational model. The effect of intensity is strongly boosted by pitch; their opposite interaction is weaker. Features capturing timbre have complex interactions between themselves depending on the experiment. It is important to note that the overall interactions observed reflect the redundancy in the computational features—e.g., intensity is encoded, to some extent, in the spectrogram, and thus bandwidth, therefore these features tend to spike together, leading to likely interactions between them. The observed effects should be interpreted within the context of the feature levels tested in the human experiments.

The probabilistic saliency output of the model can function as a discrete deviance detection mechanism by mapping the saliency scores to a binary classification. The performance of the model as a deviance detector is evaluated with an ROC curve, which maps the discrimination ability of the classifier as true detections (“hit rate”) against false detections (“false alarm”). Detection rates are computed for every possible threshold in the range [0, 1] with a step size of 0.001. The resulting ROC curves of the model (with weights from training all experimental stimuli simultaneously) are shown in Figure 6D, along with each subject's performance as mapped onto the ROC space. We select optimal thresholds on the curve based on the *d*′ metric, which quantifies the discrimination ability of the classifier at each location of the ROC space. The average human *d*′ values obtained from our psychoacoustic experiments are: I: 3.61, II: 1.88, III: 2.67. Selecting the thresholds for each experiment that produce the closest hit rate to human results, we obtain *d*′ values of I: 1.11, II: 1.20, III: 3.10. On the other hand, if the model is tuned as an absolute deviance detector (i.e., based on ground truth of deviant events), it yields *d*′ values of: I: 2.29, II: 1.72, III: 4.74. In comparison, the *d*′ values on the same stimuli run through the Kayser *et al*. saliency model (Kayser et al., 2005) are: I: 0.91, II: 0.78, III: 0.52 (scores correspond to maximum amplitude of the saliency map, parallel to our definition of the saliency score in this study). Moreover, unlike the static nature of previous auditory saliency models, the current computational model reveals a temporal build-up behavior similar to that observed in the speech experiment (Figure 2B). The model *d*′ values corresponding to the four quadrants are: 2.91, 3.10, 3.21, 3.21, illustrated in Figure 6B.

## 4. Discussion

Results from our perceptual experiments reveal an intricate auditory saliency space that is multidimensional and highly interconnected. Some of the observed interactions are not unique to the current study; but have been reported in other contexts of detection, classification and discrimination tasks (Melara and Marks, 1990; Moore, 2003; Allen and Oxenham, 2013). The current work paints a more complete picture of the non-symmetric nature of interactions in the context of complex dynamic scenes. Each of the probed auditory attributes (pitch, timbre and intensity) is a complex physical property of sound that likely evokes several neural processing streams and engages multiple physiological nuclei along the auditory pathway. It remains to be seen whether the nature of interactions reported here reflects intrinsic neural mechanisms and topographies of feature maps in the sensory system; or reveals perceptual feature integration processes at play in auditory scene analysis.

The study of bottom-up auditory attention appears to be intimately linked to processes of auditory scene perception and formation of auditory objects. The current work argues for a strong link between tracking statistics of an auditory scene and elicitation of deviance signals that flag salient sounds as aberrant events that would be attention grabbing. This process builds strongly on the notion of predictive inference, and frames the analysis of auditory scenes and selecting events of interest via predictive interpretations of the underlying events in the scene. The saliency processes presented here could be interpreted as signals for marking the reset of the grouping process in auditory streaming; flags of deviant events within an existing perceptual stream; or indicators of initiation of a new auditory object which does not fit within the expected fluctuations of the ongoing stream. Such notion is intimately linked to the concept of regularity tracking as an underlying mechanism for perception in auditory scenes (Winkler et al., 2009), with accumulating evidence that strongly tie predictive models of sensory regularity and stream segregation (Bendixen et al., 2010; Andreou et al., 2011). Some of the computational primitives presented in the current model could be seen as a shared neural infrastructure that mediates regularity tracking in a sensory-driven way (Rahne and Sussman, 2009), both to provide putative interpretations of the auditory scene as well as flag pertinent events of interest (guided by bottom-up attentional processes). The strong effect of timing on perception of saliency demonstrated by our pyschoacoustical and computational findings further hints to ties between the inference process observed here and the phenomenon of build-up of auditory streaming (Bregman, 1978; Anstis and Saida, 1985; Micheyl et al., 2005; Haywood and Roberts, 2010) or its perceptual stability (Pressnitzer et al., 2008; Kondo et al., 2012).

The model presented here is a formal implementation of the concept of regularity tracking and deviance detection in the context of dynamic scenes. These concepts have often been linked to studies of auditory attention, though the causal relationship between attention and representations of regularity is still a matter of debate (Sussman et al., 2007). The physiological bases of deviance detection is commonly probed using mismatch negativity (MMN) (Picton et al., 2000), a neural marker that emerges as the difference between responses to the “standard” and “deviant” in a stimulus often in an oddball paradigm (Winkler, 2007). The underlying mechanisms eliciting this negativity have been attributed to a potential role of memory (Naatanen et al., 1978; Garagnani and Pulvermuller, 2011) or caused by neural habituation to repeated stimulation (May and Tiitinen, 2010). A unifying framework for these mechanisms has been proposed in theories of Bayesian inference (Winkler, 2007; Bendixen et al., 2012; Lieder et al., 2013). The premise is based on the notion that the “Bayesian brain” continuously makes likelihood inferences about its sensory input, conceivably by generating predictions about upcoming stimuli (Friston, 2010). Predictive coding is arguably the most biologically plausible mechanism for making these inferences, implicating a complex neurocircuitry spanning sensory, parietal, temporal and frontal cortex (Bastos et al., 2012). The computational framework presented in this study follows the same predictive coding premise to model mechanisms of bottom-up auditory attention. It formalizes key concepts that emerge from our perceptual findings; namely: use of dynamical system modeling to capture the behavior of the acoustic scene and its time-dependent statistics; tracking the state of the system over time to infer evolution of sound streams in the scene; generating expectations about stimuli that adapt to the fidelity of sensory evidence and lead to a build-up effect of saliency detection accuracy; multidimensional mapping of sensory data that enables integrated cross-channel deviance detection while accounting for complex interactions in this multi-feature space. Kalman filtering is a natural fit for modeling such behavior. It provides an online tool for tracking evolution of states of a dynamical system that reflect past behavior and expected trajectory of the system. In many respects, the Kalman filter is equivalent to iterative Bayesian filtering under certain assumptions (Chen, 2003), and can be implemented using biologically plausible computations in neural circuits (Szirtes et al., 2005; Linsker, 2008). However, the Kalman formulation remains a linearized approximation of the dynamic behavior of acoustic scenes. More suitable frameworks such as particle filtering (Ristic et al., 2004) or recurrent Bayesian modeling (Mirikitani and Nikolaev, 2010) as well as non-Bayesian alternatives based on Volterra system analysis (Korenberg and Hunter, 1996) need to be investigated to provide a more complete account of the inference process in everyday acoustic scenes.

The use of predictive coding in the model takes a different direction from common modeling efforts of saliency in other modalities, particularly in vision. There is an abundance of models that implement concepts of stimulus-driven visual attention in which the theory of contrast as measure of conspicuity of a location in a visual scene plays a crucial role (see Borji and Itti, 2013 for a recent review). These models vary in their biological plausibility and anatomical fidelity to the circuitry of the visual system, and differ in their focus on sensory-based vs. cognitive-based processes for attentional bias of visual information. Very few models have explored the role of Bayesian inference in modeling visual saliency. Recent work has started exploring the notions of expectation, predictability and surprise as a conceptual framework for visual saliency (Itti and Baldi, 2006; Bruce and Tsotsos, 2009; Chikkerur et al., 2010). While the notion of “prediction” or predictive coding is implicit in these models, they incorporate many of its conceptual elements and could rely on the canonical circuits of predictive coding that are pervasive throughout processing stages of visual cortex (Bastos et al., 2012; Spratling, 2012). In parallel, there is greater interest in physiologically probing change detection in vision, particularly its event-related brain potential (ERP) component of visual mismatch negativity (vMMN). vMMN has been described in a number of recent studies over the last decade (see Kimura, 2012 for a review), though it has only been probed using temporal sequences and changing stimuli. Recent findings have also reported somatosensory magnetic mismatch negativity (MMNm) (Akatsuka et al., 2007) and olfactory mismatch negativity (oMMN) (Sabri et al., 2005), suggesting that MMN is a common framework for change detection across sensory modalities. The ubiquity of deviance detection in sensory cortex raises the question of commonalities among different senses in attentional selection mechanisms; or whether the parallels between audition and other senses are limited to change detection in dynamic sequences and time-dependent signals. Moreover, it remains to be seen whether saliency processes can be fully accounted for by stimulus features that induce pop-out or whether the complex interaction between sensory attributes, global proto-objects, semantic guidance and top-down attentional feedback is necessary to complete our understanding of bottom-up attention.

### Conflict of interest statement

The authors declare that the research was conducted in the absence of any commercial or financial relationships that could be construed as a potential conflict of interest.
